# Functional Connectivity of Cognitive Brain Networks in Schizophrenia during a Working Memory Task

**DOI:** 10.3389/fpsyt.2017.00294

**Published:** 2017-12-22

**Authors:** Douglass Godwin, Andrew Ji, Sridhar Kandala, Daniel Mamah

**Affiliations:** ^1^Department of Psychiatry, Washington University School of Medicine, St. Louis, MO, United States

**Keywords:** schizophrenia, functional connectivity, working memory, task-based, network, frontoparietal

## Abstract

Task-based connectivity studies facilitate the understanding of how the brain functions during cognition, which is commonly impaired in schizophrenia (SZ). Our aim was to investigate functional connectivity during a working memory task in SZ. We hypothesized that the task-negative (default mode) network and the cognitive control (frontoparietal) network would show dysconnectivity. Twenty-five SZ patient and 31 healthy control scans were collected using the customized 3T Siemens Skyra MRI scanner, previously used to collect data for the Human Connectome Project. Blood oxygen level dependent signal during the 0-back and 2-back conditions were extracted within a network-based parcelation scheme. Average functional connectivity was assessed within five brain networks: frontoparietal (FPN), default mode (DMN), cingulo-opercular (CON), dorsal attention (DAN), and ventral attention network; as well as between the DMN or FPN and other networks. For within-FPN connectivity, there was a significant interaction between *n*-back condition and group (*p* = 0.015), with decreased connectivity at 0-back in SZ subjects compared to controls. FPN-to-DMN connectivity also showed a significant condition × group effect (*p* = 0.003), with decreased connectivity at 0-back in SZ. Across groups, connectivity within the CON and DAN were increased during the 2-back condition, while DMN connectivity with either CON or DAN were decreased during the 2-back condition. Our findings support the role of the FPN, CON, and DAN in working memory and indicate that the pattern of FPN functional connectivity differs between SZ patients and control subjects during the course of a working memory task.

## Introduction

Schizophrenia (SZ) is among the most disabling brain disorders. Clinically, patients are characterized by the presence of hallucinations, delusions, disorganization, and/or negative symptoms. The majority of affected individuals also experience pervasive cognitive deficits, including those involving working memory function (1–3), which are a major cause of poor social and vocational outcomes (4–6). Cognitive deficits typically are present before illness onset and persist throughout the course of the disorder (7, 8).

Working memory is a limited capacity memory system involved in temporarily storing and manipulating information. Deficits in the working memory system in SZ are commonly linked to abnormal functioning of the dorsolateral prefrontal cortex (DLPFC) (9, 10). A meta-analysis and selective review of functional neuroimaging studies of working memory in SZ found some differences across studies, with several showing decreased activation of bilateral DLPFC, rostral prefrontal cortex, and right ventrolateral/insular cortex in SZ patients compared to controls (11, 12). Several other studies showed hyperactivation in the left frontal pole, right DLPFC, and the anterior cingulate in SZ patients (11, 12). There have been fewer resting-state functional connectivity studies conducted; however, associations between working memory performance and prefrontal cortical dysconnectivity in SZ patients have been found (13–15). Others have reported that functional connectivity within the frontoparietal network (FPN), which is involved in cognitive control (16), and the task-negative default mode network (DMN) (17) was inversely related to working memory performance.

Unlike resting-state imaging studies, task-based studies can provide direct insight into brain functional connectivity involved in working memory since imaging occurs during task performance. Most task-based SZ studies have investigated functional connectivity of the FPN, and to a lesser extent the DMN (18). Results have been variable, at least partly attributable to differences in imaging analyses methods and tasks administered. Repovs and Barch reported that functional connectivity within both the FPN and DMN, as well as between these networks and the cingulo-opercular network (CON), was modulated by working memory load in both control and SZ participants (19). Reduced FPN functional connectivity and decreased prefrontal activation has been found in SZ patients during a working memory task (20, 21). Abnormal prefrontal cortical functional connectivity with the striatum (22), anterior cingulate (21), or hippocampus (23) has also been reported in SZ during with task performance. Despite multiple reports of reduced FPN connectivity with working memory in SZ, normal (15, 24) and increased (25) task-based prefrontal connectivity also been reported. Other authors found DMN dysconnectivity in SZ during a working memory task, including a failure of medial frontal cortical deactivation (26) or aberrant hippocampal coupling with the DMN (25).

The current study investigates FPN and DMN functional connectivity during the *n*-back task in SZ and control participants, as well as connectivity with other known cognitive brain networks: the cingulo-opercular (CON), dorsal attention (DAN), and ventral attention (VAN). Our study contributes to the literature on working memory task-based functional connectivity in SZ, by investigating multiple cognitive networks derived using a novel network-based cortical parcelation approach (27). Additionally, our study comprises of the only existing connectivity dataset from SZ patients obtained using the customized scanner of the Human Connectome Project (HCP) (28), a multi-site National Institutes of Health effort aimed at mapping the neural pathways of the healthy brain. Our scanning protocol and *n*-back task were also identical to that of the HCP. We hypothesized that decreased DMN and increased FPN functional connectivity during an *n*-back task would be attenuated SZ, especially with a high cognitive load.

## Materials and Methods

### Participants

Demographic and clinical information for all participants are shown in Table 1. The Institutional Review Board at the Washington University School of Medicine approved all study protocols. Participants provided written, informed consent prior to participation in the study. Imaging data were collected from 33 healthy control (CN) and 27 SZ participants, aged 18–30 years recruited from the greater St. Louis area. Four participants were excluded from analysis due to incomplete data acquisition (two CN and two SZ). SZ participants were outpatients and met DSM-IV criteria for SZ, as assessed by both the Structured Clinical Interview for the DSM-IV Axis I Disorders (SCID-I) (29) and a clinical evaluation by a research psychiatrist (D.M.). All potential participants were excluded if they met DSM-IV criteria for substance dependence or severe/moderate abuse in the past 6 months, were at the time of recruitment clinically unstable (i.e., significantly sedated or unable to follow instructions) or had a history of head injury or loss of consciousness. Exclusion criteria for CN included a lifetime history of DSM-IV psychotic or mood disorders.

**Table 1:** Demographics and clinical information.

Characteristics	CON (*n* = 31)	SCZ (*n* = 25)	Test statistic	*p*-Value
Mean age (SD)	24.4 (2.8)	24.9 (3.3)	*t*_(54)_ = 0.641	0.53
Sex (%)			$\chi_{\left(1\right)}^{2}=5.90$	0.02
Males	15 (48.4)	20 (80.0)		
Females	16 (51.6)	5 (20.0)		
Race (%)			$\chi_{\left(4\right)}^{2}=9.31$	0.05
Asian	5 (16.1)	1 (4.0)		
Black	7 (22.6)	14 (56.0)		
Hispanic	0	0		
White	18 (58.1)	8 (32.0)		
Multiracial	1 (3.2)	2 (8.0)		
Handedness (%)			$\chi_{\left(1\right)}^{2}=0.05$	0.83
Left	3 (9.7)	2 (8.0)		
Right	28 (90.3)	23 (92.0)		
History of Use Disorder (%)^a^				
Alcohol	3 (9.7)	5 (20.0)	$\chi_{\left(1\right)}^{2}=1.20$	0.27
Cannabis	3 (9.7)	6 (24.0)	$\chi_{\left(1\right)}^{2}=2.11$	0.15
Stimulant	0	0	–	–
Opioid	0	0	–	–
Cocaine	0	0	–	–
Hallucinogen	0	0	–	–
Lifetime Psychotropic Medication (%)				
Typical neuroleptic	0	12 (48.0)	$\chi_{\left(1\right)}^{2}=18.94$	<0.001
Atypical neuroleptic	0	25 (100.0)	$\chi_{\left(1\right)}^{2}=56.00$	<0.001
SSRI	3 (9.7)	16 (64.0)	$\chi_{\left(1\right)}^{2}=18.22$	<0.001
Other antidepressants^b^	0	0	–	–
Stimulant	1 (3.2)	0	$\chi_{\left(1\right)}^{2}=0.82$	0.365
Mood stabilizer	1 (3.2)	11 (44.0)	$\chi_{\left(1\right)}^{2}=13.67$	<0.001
Benzodiazepines	1 (3.2)	10 (40.0)	$\chi_{\left(1\right)}^{2}=11.86$	0.001
Anticholinergic	0	8 (32.0)	$\chi_{\left(1\right)}^{2}=11.57$	0.001
None	28 (90.3)	0	$\chi_{\left(1\right)}^{2}=39.140$	<0.001
Duration of Illness—months (SD)	N/A	70.9 (45.6)	–	–
Symptom domains (SD)				
SAPS^c^				
Positive symptom subscales	0	3.68 (2.8)	*t*_(54)_ = 7.388	<0.001
Disorganized symptom subscales	0	1.04 (1.5)	*t*_(54)_ = 3.907	<0.001
SANS^d^				
Negative Symptom Subscales	0.55 (1.3)	6.2 (3.0)	*t*_(54)_ = 5.036	<0.001

*^a^Other than for nicotine use disorder, participants did not meet criteria for a use disorder in the last 6 months*.

*^b^Refers to antidepressants other than selective serotonin reuptake inhibitors (SSRI)*.

*^c^Maximum possible score on the Structured Assessment of Positive Symptoms (SAPS) is 16*.

*^d^Maximum possible score on the Structured Assessment of Negative Symptoms (SANS) is 20*.

### Cognitive Assessment

Prior to scanning, participants completed a computer-based cognitive assessment, the University of Pennsylvania Computerized Neurocognitive Battery (Penn-CNB) (30). Cognitive scores were determined as previously (31, 32), based on the results of working memory and attention related cognitive tasks including the Penn Abstraction, Inhibition, and Working Memory Task (AIM) (33), Penn Continuous Performance Test-Number and Letter Version (CPT) (34), and Letter-N-Back (LNB) (35). A composite working memory performance score was calculated by *z*-scoring the true positive rate for the LNB 1-back and 2-back conditions and summing with the *z*-score of the true positive rate of the working memory portion of the AIM task. An attention score was generated by *z*-scoring the accuracy (all correct responses/all trials) of the CPT task results.

### Behavioral Paradigm for Task fMRI

The *n*-back task and procedure used has been previously described (36). Briefly, the task was practiced outside the scanner prior to beginning the scan session. Task instructions were presented and explained again in the scanner prior to the beginning of each task run. Two runs per participant were obtained, each 5:01 mins long and containing eight task blocks, split evenly between 0-back and 2-back conditions. Each block lasted 25 s and consisted of 10 2.5-s trials. Interspersed between task blocks were 15-s fixation blocks. Stimuli were presented using the E-prime (2.0.10.242) presentation software and back projected onto a screen outside of the scanner. Participants viewed this screen *via* a mirror attached to the head coil inside the scanner. Four types of stimuli were shown: faces, places, tools, and body parts. Task blocks within each run were evenly split between each of the four stimulus types. Participants responded on each trial whether the stimulus image matched the target image using one of two right-handed button presses for “match” or “no match.” In the 0-back condition, participants were asked to respond whether each presented stimulus matched a target image presented at the start of each 0-back block. The 2-back required participants to respond whether each presented stimulus matched the image presented two trials prior to the current image.

Participants who had <70% accuracy on the 0-back portion of a run, performed an additional run to replace the lowest performance scan.

### fMRI Data Acquisition and Preprocessing

The fMRI protocol mirrored the HCP Phase 2 task functional MRI acquisition (37). These acquisitions utilized a 32 channel head coil on a modified 3T Siemens Skyra with TR = 720 ms, TE = 33.1 ms, flip angle = 52°, BW = 2,290 Hz/Px, in-plane FOV = 208 × 180, 72 slices, 2.0-mm isotropic voxels, and a multi-band acceleration factor of 8 was used for whole brain acquisitions as a means of increasing signal to noise (38). Each task session contained two runs with a right-to-left and a left-to-right phase encoding.

This preprocessing pipeline package output minimally preprocessed 4D time series data consisting of motion correction, gradient unwarping, brain-boundary-based registration of EPI to structural T1-weighted scan, fieldmap-based EPI distortion correction, non-linear (FNIRT) registration into MNI152 space and grand-mean intensity normalization. From here, the data were projected onto a surface representation and smoothing was constrained to cortical surface and subcortical gray-matter parcels as part of a “grayordinate”-based approach (39).

### fMRI Data Processing

Open source, HCP data analysis pipelines were used to process raw data, which utilized tools from FSL (5.0.9) and AFNI FreeSurfer (5.3.0) (39). To account for the potential influence of task-related evoked responses on correlations, we modeled task events using a general linear model regression (40–43). Separate predictors were included for each stimulus type (i.e., faces, tools, places, and body parts) crossed with each working memory load level (0-back, 2-back). To form these predictors, we convolved a canonical hemodynamic response function with block predictors for each appropriate trial, creating 0- and 2-back predictors for each stimulus type. Separately, we included a model of correct and incorrect responses by creating two predictors for all stimuli with correct or incorrect responses. Additional confound regressors included in the model were the global signal, average ventricle and white matter timecourses (calculated from data in the volume space), and six estimated parameters of motion and their first derivatives. The resulting model consisted of predictors for each stimulus by *n*-back level (0- and 2-back), correct responses, incorrect responses, and confound regressors described above. The residuals of this regression were then specified for the five specified networks (and their constituent parcels) using the existing 333-node parcelation scheme described by Gordon et al. (27). Briefly, the Gordon parcelation scheme involved boundary-map generation identifying transitions in resting-state correlations across the cortical surface, in a previously described dataset (27). The 333-node (parcel) assignments were grouped into functional networks using a process that involved community assignments based on the maximization of within-community random walks in a functional connectivity matrix, as well as harmonization with previously reported cortical network locations.

**Figure 1:**
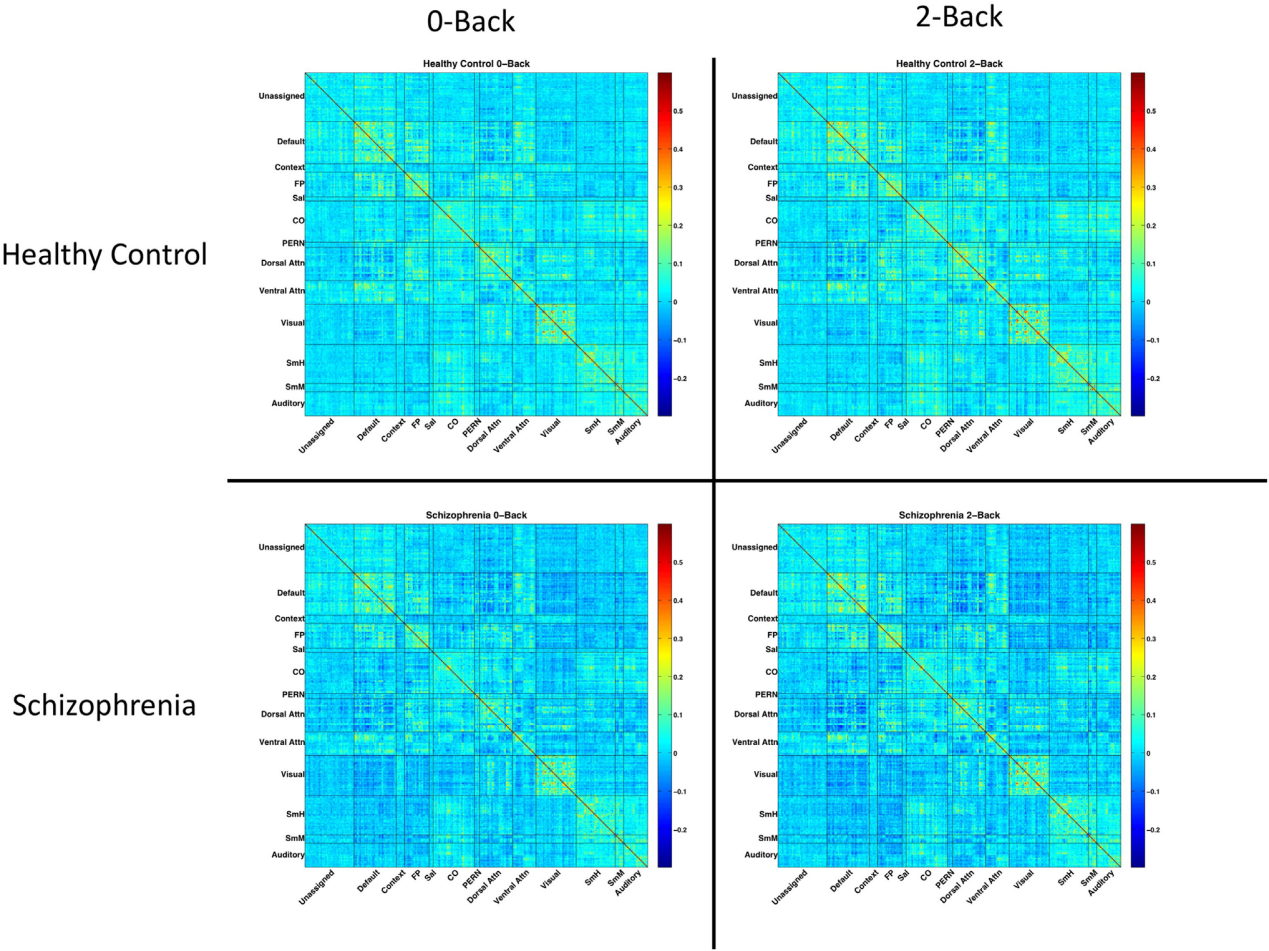
Network matrices for each diagnostic group and *n*-back condition. Each matrix is averaged across all relevant subjects and organized based on a 333-node boundary-map parcelation, detailed in Gordon et al. (27). Warmer colors indicate stronger connections. Cooler colors indicate weaker connections.

Residuals were temporally masked, for each *n*-back level, at time points corresponding to correct trials. To account for potential spurious correlations produced by high-motion frames (44, 45), time points corresponding to an observed frame-wise displacement of 0.7 mm or greater were excluded (46). 0.7 mm was chosen as the threshold to maximize the number of usable frames for analysis. Mean (SD) number of frames dropped were 0.9 (2.0) for healthy controls, and 5.0 (11.3) for SZ patients. The result was a timecourse, for each *n*-back condition, masked to include only correct trials and excluding high-motion time points. Pearson correlations between parcels were calculated on these masked, residualized time courses. Fully connected, group and condition averaged network matrices are shown in Figure 1.

### fMRI Connectivity Analysis

**Figure 2:**
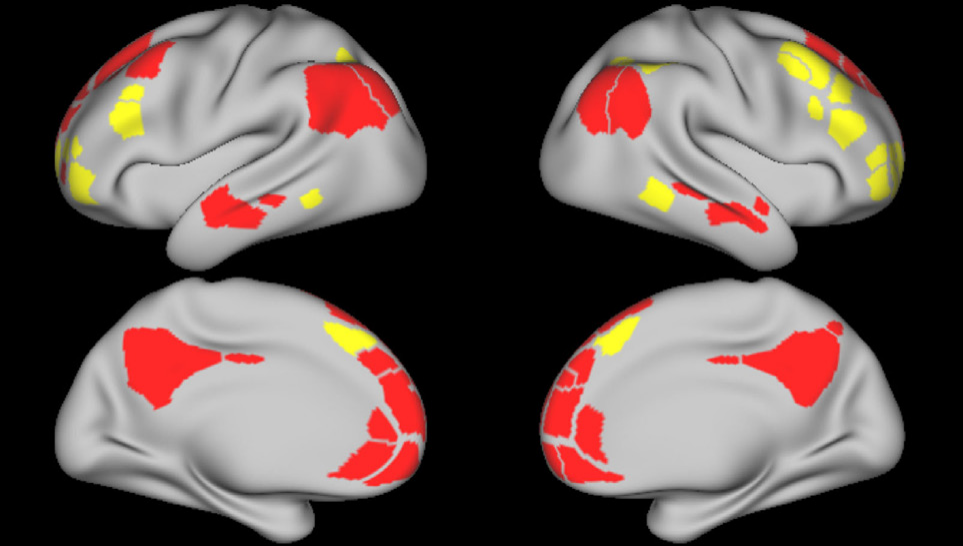
Frontoparietal and default mode network parcels. Figure depicts the frontoparietal network (yellow) and the default mode network (red) parcels, derived from boundary-map parcelation [Gordon et al. (27)]. Top images represent the left and right lateral brain surfaces, respectively. Bottom images represent the left and right medial brain surfaces. The same cortical parcels were used in both control and schizophrenia participants.

For each network of interest, we calculated the average within-network functional connectivity, with connectivity defined as the Pearson correlations between parcels comprising each network. These averages were calculated on the fully connected, symmetric, single-subject functional connectivity matrices for each *n*-back condition. Networks of interest included the DMN, FPN, CON, DAN, and VAN, derived from the parcelation scheme described by Gordon et al. (27). In addition, we calculated the average between-network connectivity for each of these networks with the FPN and DMN (see Figure 2).

### Statistical Analyses

All statistical analyses were performed using IBM SPSS Statistics, version 24 (SPSS Inc.). To test for average between- and within-network differences across *n*-back condition and group, we utilized a 2 × 2 ANCOVA, with diagnostic group (control vs. SZ; between subject) and *n*-back condition (0-back vs. 2-back; repeated-measures) as factors. All analyses included number of frames dropped due to head-motion censoring as a covariate. For within-network tests, *p-*values are Bonferroni corrected for five tests (one per network tested). For all between-network tests, *p*-values reported are Bonferroni corrected for seven between-network connectivity tests. Based on ANCOVA results, we performed follow-up analyses to determine whether network connections that showed an interaction between diagnosis and n-back condition correlated with clinical symptom severity (i.e., for positive, disorganized, and negative symptoms) or in-scanner accuracy. We also investigated correlations of network connectivities with cognitive performance (i.e., working memory and attention) outside the scanner. Connectivity variables for correlations were either the specific connectivity at either condition, or derived as the functional connectivity difference between conditions (i.e., 2-back minus 0-back). We tested the effect of typical antipsychotic drugs on connectivity at either task condition by comparing SZ patients on typical antipsychotics to those on only atypical antipsychotic drugs using a Student’s *t*-test.

## Results

### Working Memory Task Performance

Table 2 shows the in-scanner working memory task accuracy of CN and SZ participants. For both 0-back and 2-back, SZ participants had lower accuracy compared to CN. The table also indicates that working memory and attention performance outside the scanner was worse in SZ participants.

**Table 2:** Task performance.

	CON	SCZ	*t*	*p*-Value
**In-scanner—accuracy (SD)**				
0-back	0.91 (0.09)	0.76 (0.18)	4.105	<0.001
2-back	0.88 (0.07)	0.73 (0.17)	4.609	<0.001
**Outside-scanner—composite (all subjects)**				
Working memory^a^	0.39 (0.36)	−0.47 (1.29)	3.470	0.001
Attention^b^	0.74 (1.01)	−0.89 (1.89)	4.074	<0.001

*Cognitive scores were determined based on the results of working memory and attention related cognitive tasks, including the Penn Abstraction, Inhibition and Working Memory Task (AIM), Penn Continuous Performance Test-Number and Letter Version (CPT), and Letter-N-Back (LNB)*.

*^a^A composite working memory performance score was calculated by z-scoring the true positive rate for the LNB 1-back and 2-back conditions and summing with the z-score of the true positive rate of the working memory portion of the AIM task*.

*^b^An attention score was generated by *z*-scoring the accuracy (all correct responses/all trials) of the CPT task results*.

### Within-Network Group Effects

**Figure 3:**
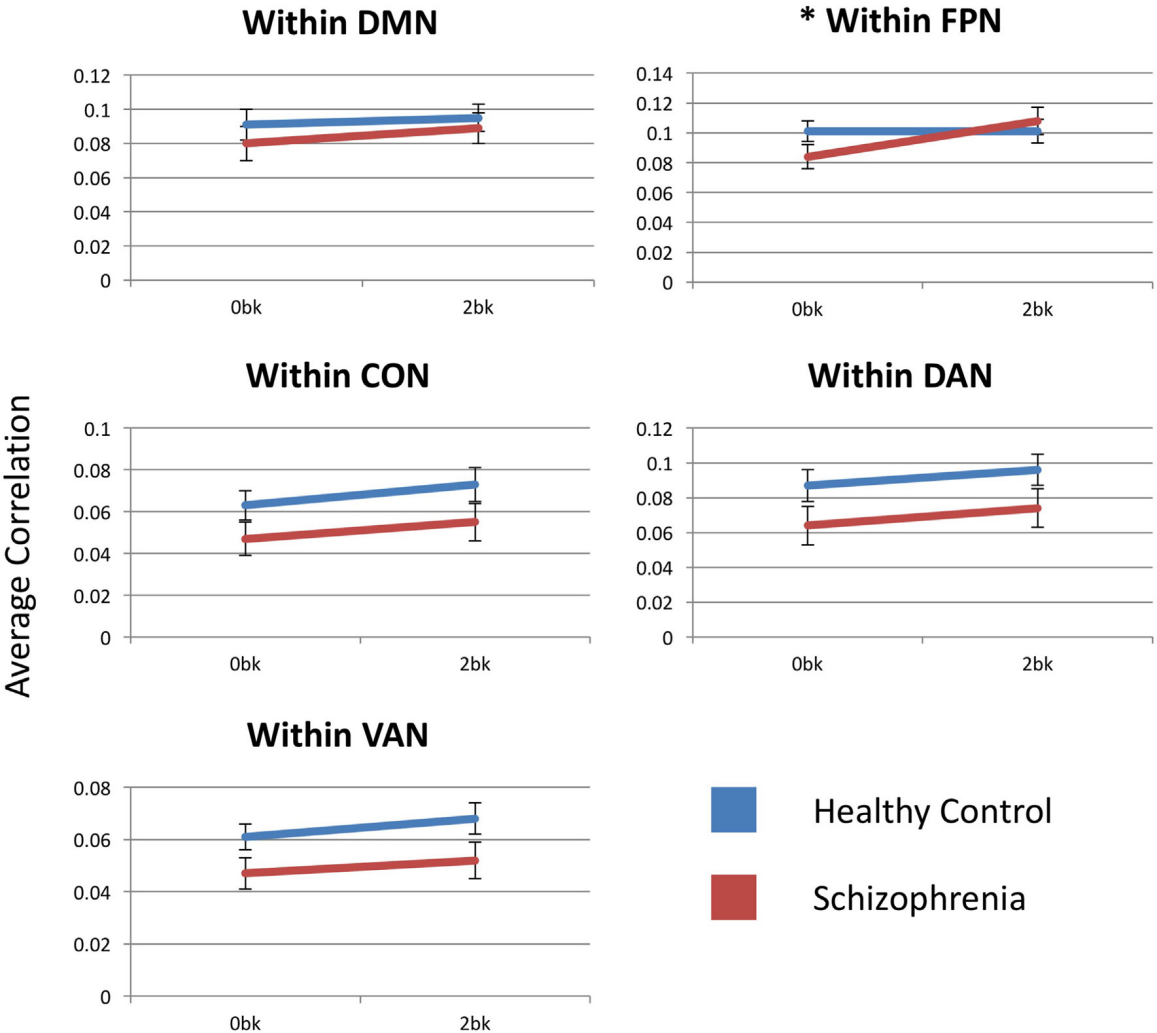
Within-network functional connectivity for the five networks assessed. Each graph presents the average within-network correlation coefficients (*y*-axis) for each network. Only the frontoparietal network showed a significant interaction between diagnostic group (Control vs. Schizophrenia patients) and *n*-back (0-back vs. 2-back). DMN, default mode network; FPN, frontoparietal network; CON, cingulo-opercular network; DAN, dorsal attention network; VAN, ventral attention network. Asterisks indicate statistical significance (*p* = 0.05) for group × condition effect. Error bars represent SEs.

Estimated marginal means by group for each within-network functional connectivity are shown in Figure 3. The FPN showed a significant main effect of *n*-back condition [*F*_(1,54)_ = 10.629, *p* = 0.01] but no main effect of diagnostic group [*F*_(1,54)_ = 0.185; *p* > 0.99]. The FPN, also showed a significant condition-by-diagnosis interaction [*F*_(1,54)_ = 9.824, *p* = 0.015], driven by decreased connectivity at the 0-back condition in SZ compared to CN participants. Unlike in CN participants, SZ participants increased within-FPN connectivity with *n*-back difficulty. *Post hoc* analyses, however, did not meet significance for group effects at 0-back [*F*_(1,54)_ = 2.028, *p* = 0.16] and 2-back [*F*_(1,54)_ = 0.265, *p* = 0.6] for within-FPN connectivity.

Within the DMN, there was no main effect of condition [*F*_(1,54)_ = 5.139, *p* = 0.14], diagnosis [*F*_(1,54)_ = 0.536, *p* > 0.99] or condition-by-group interaction [*F*_(1,54)_ = 0.713, *p* > 0.99].

There was a significant main effect of *n*-back condition for within-CON [*F*_(1,54)_ = 14.348, *p* = 0.002] and within-DAN [*F*_(1,54)_ = 9.337, *p* = 0.015] connectivity, with connectivity within both the CON and DAN increasing with *n*-back difficulty in both SZ and CN participants. There was no group effect or condition-by-group interaction for either network. Within-VAN connectivity showed only a trend level effect of *n*-back condition [*F*_(1,54)_ = 0.083, *p* = 0.09], and no group or condition-by-group effect.

### Group Effects: Between Frontoparietal and Other Networks

**Figure 4:**
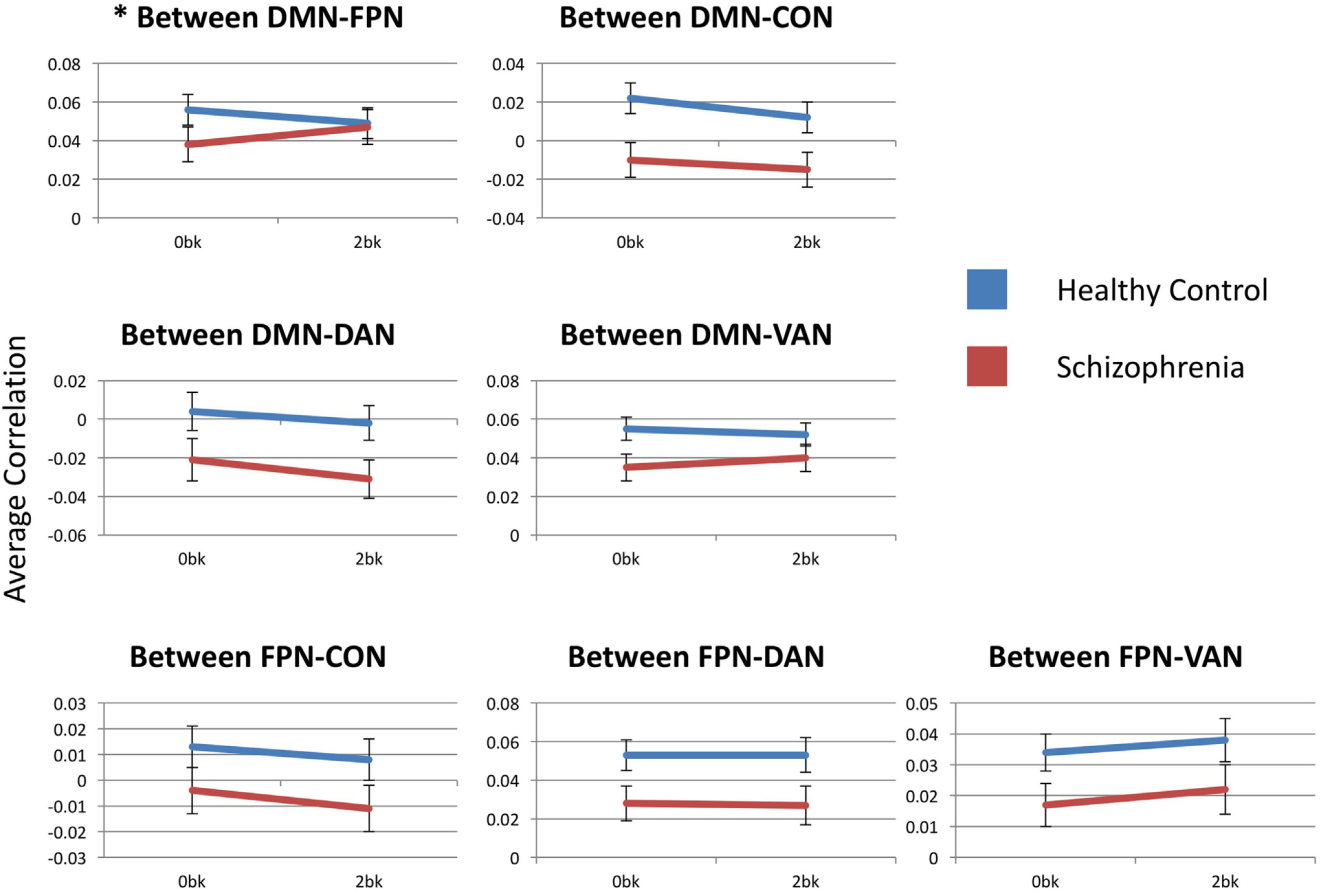
Between-network functional connectivity across group and condition. Each graph presents the average between-network correlation coefficients (*y*-axis) for each of the seven tested connections. DMN, default mode network; FPN, frontoparietal network; CON, cingulo-opercular network; DAN, dorsal attention network; VAN, ventral attention network. Asterisks indicate statistical significance (*p* = 0.05) for group × condition effect. Error bars represent SEs.

Estimated marginal mean connectivities between either FPN or DMN and other networks are depicted in Figure 4. DMN-to-FPN functional connectivity did not show a main effect of n-back condition [*F*_(1,54)_ = 0.278, *p* > 0.99] or group [*F*_(1,54)_ = 0.062, *p* > 0.99]. However, there was a significant condition-by-group interaction [*F*_(1,54)_ = 8.041, *p* = 0.042]. Notably, DMN-to-FPN connectivity with *n*-back difficulty, decreased in CN participants and increased in SZ participants. *Post hoc* analyses did not meet significance for group effects at 0-back [*F*_(1,54)_ = 2.072, *p* = 0.16] and 2-back [*F*_(1,54)_ = 0.042, *p* = 0.84].

FPN functional connectivity with either CON, DAN or VAN did not show any significant effects of condition or group, and there were no condition-by-group interactions.

### Group Effects: Between Default Mode and Other Networks

DMN-to-FPN functional connectivity results are described above. DMN-to-CON connectivity showed a significant main effect for n-back condition (*F*_(1,54)_ = 13.786, *p* = 0.003), but there was effect of group [*F*_(1,54)_ = 6.355, *p* = 0.11] or a significant condition-by-group interaction [*F*_(1,54)_ = 0.274, *p* > 0.99]. Similarly, there was a significant effect of *n*-back condition [*F*_(1,54)_ = 10.093, *p* = 0.014] for DMN-to-DAN connectivity, without group [*F*_(1,54)_ = 3.544, *p* = 0.46] or interaction [*F*_(1,54)_ = 0.741, *p* > 0.99] effects. Connectivity of the DMN with either the CON or the DAN decreased with *n*-back difficulty, in both SZ and CN groups.

There were no significant effects of n-back condition or group, or any condition-by-group interaction for DMN-to-VAN functional connectivity.

### Correlations with Cognitive Scores

Table 3 shows the correlations of in-scanner accuracy of 0-back and 2-back tests with within-FPN connectivity and DMN-to-FPN connectivity, in both subject groups. Accuracy at 0-back correlated with DMN-to-FPN connectivity significantly (*r* = 0.41; *p* = 0.04) and at a trend level with within-FPN connectivity (*r* = 0.4; *p* = 0.051). Accuracy at 2-back did not correlate significantly with either FPN connectivity.

**Table 3:** Clinical and cognitive relationships with frontoparietal network connectivities.

	SCZ (FPN)	SCZ (FPN-DMN)	CON (FPN)	CON (FPN-DMN)
**In-scanner—accuracy**				
0-back	0.40 (0.05)*	0.41 (0.04)**	0.25 (0.18)	0.19 (0.30)
2-back	0.32 (0.12)	0.05 (0.81)	0.25 (0.17)	0.24 (0.19)
**Positive symptoms**				
0-back	0.24 (0.25)	0.17 (0.41)	N/A	N/A
2-back	0.10 (0.62)	0.26 (0.22)	N/A	N/A
**Negative symptoms**				
0-back	−0.12 (0.58)	−0.19 (0.36)	0.21 (0.26)	0.25 (0.18)
2-back	0.08 (0.71)	−0.03 (0.87)	0.14 (0.47)	0.09 (0.66)
**Disorganized symptoms**				
0-back	0.09 (0.67)	−0.06 (0.79)	N/A	N/A
2-back	0.05 (0.81)	−0.08 (0.69)	N/A	N/A

*Values are given in R (p value)*.

**p < 0.05, uncorrected. *p < 0.10, uncorrected.

We also investigated the relationship of connectivities with working memory or attention performance outside the scanner. Across groups, significant correlations were observed for working memory performance with the within-FPN functional connectivity difference (*r* = −0.3; *p* = 0.04) and a trend level correlation with the DMN-to-FPN functional connectivity difference (*r* = −0.29; *p* = 0.06). However, there was no significant relationship when only SZ participants were analyzed. Attention performance did not correlate significantly with either the FPN or DMN-to-FPN connectivity difference, in either all participants or SZ participants.

### Correlations with Clinical Ratings

We tested the relationship of FPN connectivity and DMN-to-FPN connectivity, with clinical symptoms (i.e., positive, disorganized, and negative symptoms). Across groups, significant correlations were observed for negative symptoms with the within-FPN functional connectivity difference (*r* = 0.35; *p* = 0.01), and a significant correlation of positive symptoms with the DMN-to-FPN functional connectivity difference (*r* = 0.30; 0.03).

As seen in Table 3, there were no significant relationships of any clinical symptom with FPN connectivities at 0-back or 2-back in either group.

### Medication Effects

We investigated the effects of the major classes of antipsychotics among SZ patients, by comparing FPN connectivities in those on typical antipsychotics (*N* = 12) and those on only atypical antipsychotics (*N* = 13). There were no significant effects observed for either within-FPN connectivity (0-back: *F* = 1.1, *p* = 0.3; 2-back: *F* = 1.4, *p* = 0.2) or DMN-to-FPN connectivity (0-back: *F* = 0.02; *p* = 0.9; 2-back: *F* = 1.3; *p* = 0.3).

## Discussion

A goal of the current study was to investigate brain functional connectivity changes with increasing difficulty on an *n*-back working memory task (i.e., from 0-back to 2-back tests). In addition, we studied functional connectivity differences between SZ patients and healthy participants while performing these tasks. Connectivity was investigated across five cognitive network that were expected to be relevant to working memory. Our neuroimaging dataset was unique, being the only one obtained from SZ patients using the customized HCP primary scanner, located at Washington University.

### Network Functional Connectivity and Working Memory Load

Our study found that across subjects from both groups, functional connectivity within the frontoparietal (FPN), cingulo-opercular (CON), and dorsal attention (DAN) network increased with cognitive load. However, increased connectivity within the FPN was driven by the SZ group, with control participants showing similar connectivity during the 0-back and 2-back tests. This was in contrast to within the CON and DAN, where connectivity in both control and SZ participants increased with cognitive load. The CON and DAN also showed increased decoupling from the task-negative default mode network (DMN) with increased cognitive load, suggesting that the CON and DAN are involved in task processing. Both the CON and DAN have known roles in cognition. The CON includes brain regions that include the anterior insula/operculum and dorsal anterior cingulate cortex. Unlike the FPN that has been ascribed roles in the initiation and modulation of cognitive control abilities, the CON is thought to facilitate the maintenance of task-relevant goals and the incorporation of error information to adjust behavior (47, 48), and are co-activated during cognitive control tasks (49, 50). On the other hand, the DAN is an intrinsic network which comprises of regions of the intraparietal and superior frontal cortices, and has been reported to be involved in top-down, goal-directed selection deployment of attention (51). Our findings indicate that carrying out a working memory task requires increased neural processing within these three brain networks (FPN, CON, and DAN). The results further suggest that increased task-related neural processing within the FPN may be more important for those with psychopathology, than for healthy individuals, possibly due to decreased efficiency of the other cognitive networks.

Our results are supported by reports from other studies. While negative findings have been reported (19), the CON has been found to flexibly link with other brain networks during cognitive tasks, and to be involved in a broad range of cognitive processes (52). Functional connectivity or increased coherence within the CON have also been reported to increase with tasks requiring more tonic alertness (53), word recognition (54), visuospatial attention and episodic memory, memory encoding of speech in noise (55) and slow reveal task (56). In addition, general cognitive ability has been linked to the local and global efficiency of the CON in both control and SZ participants, and those with psychotic-like experiences (57, 58). It has been suggested that the CON plays a more downstream role in cognitive control, compared to the FPN, possibly associated with output gating of memory. For example, Wallis et al. reported that when spatial cues occurred indicating the relevant item in a working memory array, the FPN was activated following the cue (59). However, when cues occurred during the maintenance period, FPN activation was transient and succeeded by CON activation. In our study, the association of increased within-DAN connectivity with cognitive load was consistent with the role of the DAN in controlling the spatial orientation of attention. Activation patterns of specific DAN regions have been found to predict both verbal and visual working memory load (60). Also, increased DAN activation has also been reported with increasing short-term memory load, in contrast to the ventral attention network activity, which was decreased (61).

### Group Differences in Task-Based Functional Connectivity

We found two significant connectivity differences between SZ patients and control participants in our study. First, there was decreased within-FPN connectivity during the 0-back condition in SZ patients, compared to controls; however, during the 2-back condition, FPN connectivity was similar across groups. Second, connectivity between the FPN and the default mode network (DMN) decreased with greater working memory load in healthy participants, but increased in SZ patients. The connectivity of other cognitive networks studied tended to be lower in SZ than controls, consistent with a generalized hypoconnectivity that has been widely described (62, 63), although these group differences did not meet statistical significance. These findings suggest that in SZ patients, the FPN is hypoconnected during with a lower cognitive load, but patients are capable of achieving the needed connectivity to process more complex tasks. At the same time, in patients, the FPN does not uncouple from the DMN with high cognitive load as it does in healthy participants; rather, they become more strongly connected. While speculative, such a finding may suggest that in SZ patients, low connectedness of the FPN at low cognitive load or at rest, may be overcome by recruitment of compensatory pathways with increasing task complexity.

Our study results support the role of FPN cortical regions, including the DLPFC, in SZ. Meta-analytic and review studies of working memory have generally found reduced activation of the DLPFC in most studies of SZ patients compared to healthy controls (11, 12, 64). Group differences in other areas are also seen, including increased activation of anterior cingulate and the left frontal pole (11). This have been suggested to represent a dysfunctional brain network supporting working memory in SZ, which involves impaired attention control by the DLPFC, with associated increases in error monitoring involving the anterior cingulate (11), functioning patients also showed increased activation in some prefrontal regions (65). Results of studies involving FPN functional connectivity in SZ have been variable, and may be related to methodological differences and biology heterogeneity of the disorder. Nielsen et al. reported that the increased FPN connectivity modulated by working memory, was decreased in first-episode SZ patients (66). Repovs and Barch however, found increased FPN connectivity in both control and SZ populations with increased working memory load (19). Similar to our findings, Eryilmaz et al. reported normalization of FPN functional connectivity during a working memory task, despite impaired resting-state connectivity (24). These authors further found that working memory deficits were related to limbic and thalamic dysconnectivity and altered connectivity between FPN and the thalamus.

The underlying neurobiology of decreased functional connectivity in SZ with working memory may be related to decreased integrity of key white matter tracts within the brain. Functional connectivity is not isomorphic with structural connectivity (at least in terms of single synapse connections) and thus one cannot directly interpret alterations in functional connectivity as reflecting alterations in structural connectivity. White matter abnormalities in SZ have, however, been well described from diffusion studies (67, 68), including decreased white matter integrity in the superior longitudinal fasciculi, the main frontal-parietal white matter connection. Superior longitudinal fasciculi integrity has also been related to working memory performance in SZ (69). Such findings are consistent with an abnormal neurodevelopment in SZ, with regional synaptic deficit and intact long-distance connections (70, 71). Our findings point to a need to more directly examine the degree to which changes in functional connectivity are reflective of changes in white matter integrity in SZ.

### Limitations

Despite the relevance of the results presented, our study does have some limitations that may influence our findings. First, as with other functional connectivity studies, potential motion-related effects may have affected data quality. While our motion-censoring is expected to minimize such influences, motion-related differences across groups may have confounded our results. The amount of data available could also have influenced the reliability of our connectivity results (72–74). While the data volume used in our study is comparable to other task-related connectivity studies in SZ, it is possible that the available data after motion-censoring may have become insufficient to accurately estimate functional connectivity. Second, the network parcelation scheme used in our study was derived from resting-state data. While the spatial locations of task-based networks in the brain largely overlap with that of resting-state networks, differences may also be present. Some differences in the spatial location of networks are also expected across working memory conditions. Thus, no parcelation scheme can be universally applicable. This should be considered in interpreting results; as cortical parcels may not fully delineate each specified network in each individual. Potential inaccuracies in network parcels would, however, be expected to affect individuals in each group similarly. Future studies using alternative parcelation schemes will be useful to validate our findings. Thirdly, our study was not designed to investigate differences in regional blood oxygen level dependent activation during the working memory task between SZ and control groups. Such differences, however, would present a more complete picture of the brain regions recruited during task performance, and could influence the interpretation of the connectivity findings. For instance, decreased activation of DLPFC (a major component of the FPN) in SZ during working memory, as seen in several studies (11, 12, 64), may contribute to the observed decreased FPN connectivity in our study. Future investigations would, therefore, benefit from including information on brain activation alongside connectivity results. Finally, the role of antipsychotic treatment of SZ also may influence neuroimaging findings during working memory tasks. For example, Wolf et al. (75) found improved frontotemporal function after several weeks of antipsychotic treatment, together with improved working memory performance in SZ patients. Others report normalization of frontal cortical activation only specific antipsychotic drugs, including aripiprazole (76, 77), Quetiapine (78) or risperidone (79). Normalizing of functional dysconnectivity in SZ participants have also been reported (80, 81). Thus, it is likely that functional findings in SZ patients are underestimated, and medications likely confound the understanding of the pathophysiology of working memory deficits in SZ patients. Studies involving medication naïve patients, unmedicated high-risk individuals and longitudinal treatment studies would, therefore, shed light on specific medication effects on task-based connectivity.

## Conclusion

In summary, our study focused on the connectivity of five key cognitive networks, which were defined using a validated cortical network parcelation approach, with imaging data obtained using the HCP primary scanner. We found decreased FPN functional connectivity and FPN-to-DMN functional connectivity during the 0-back condition, relative to the 2-back condition, in SZ patients compared to controls. In addition, increased CON and DAN functional connectivity was found with increased working memory load in both control and SZ participants. Normalization of FPN connectivities with increasing cognitive load, suggests that FPN dysconnectivity in SZ occurs mainly with tasks requiring low effort. These findings provide evidence for the interplay of these cognitive networks in working memory performance and for abnormal cognitive processing in SZ, supporting results from other studies. Future studies would be important to investigate the specificity of our findings to working memory tasks, and the role of other brain networks.

## Ethics Statement

This study was carried out in accordance with the recommendations of the Institutional Review Board (IRB) of Washington University, St. Louis with written informed consent from all subjects. All subjects gave written informed consent in accordance with the Declaration of Helsinki. The protocol was approved by the Institutional Review Board (IRB) of Washington University.

## Author Contributions

DG wrote the first draft of the manuscript and conducted the majority of the analyses. AJ and SK conducted some of the analyses. DM planned and supervised the study and analyses.

## Conflict of Interest Statement

The authors declare that the research was conducted in the absence of any commercial or financial relationships that could be construed as a potential conflict of interest.
